# Comparison of oral microbiome profiles in 18-month-old infants and their parents

**DOI:** 10.1038/s41598-020-78295-1

**Published:** 2021-01-13

**Authors:** Ryutaro Jo, Kazuma Yama, Yuto Aita, Kota Tsutsumi, Chikako Ishihara, Masato Maruyama, Kaori Takeda, Eiji Nishinaga, Ken-ichiro Shibasaki, Seiji Morishima

**Affiliations:** 1 Research and Development Headquarters, Lion Corporation, 7-2-1 Hirai, Edogawa-ku, Tokyo, 132-0035 Japan; 2 The Lion Foundation for Dental Health, 1-3-7, Honjo, Sumida-ku, Tokyo, 130-8644 Japan

**Keywords:** Microbiome, Oral microbiology

## Abstract

The onset and progress of dental caries and periodontal disease is associated with the oral microbiome. Therefore, it is important to understand the factors that influence oral microbiome formation. One of the factors that influence oral microbiome formation is the transmission of oral bacteria from parents. However, it remains unclear when the transmission begins, and the difference in contributions of father and mother. Here, we focused on the oral microbiome of 18-month-old infants, at which age deciduous dentition is formed and the oral microbiome is likely to become stable, with that of their parents. We collected saliva from forty 18-month-old infants and their parents and compared the diversity and composition of the microbiome using next-generation sequencing of 16S rRNA genes. The results showed that microbial diversity in infants was significantly lower than that in parents and composition of microbiome were significantly different between infants and parents. Meanwhile, the microbiome of the infants was more similar to that of their mothers than unrelated adults. The bacteria highly shared between infants and parents included not only commensal bacteria but also disease related bacteria. These results suggested that the oral microbiome of the parents influences that of their children aged < 18 months.

## Introduction

Dental caries and periodontal disease are the most prevalent diseases in the world^1,2^. These are infectious diseases caused by oral bacteria and are associated with a shift from symbiotic microbiota to dysbiosis^3,4^. After birth, normal oral microbiome is formed, which has a symbiotic relationship with the host^4^. Therefore, it is important to understand the formation process of normal oral microbiome and to maintain its state for the prevention of disease. However, the process of normal oral microbiome formation after birth and the factors that influence its formation remain unclear.

At birth, few bacteria are present in the oral cavity^5,6^. Diversity of the oral microbiome increases over time^7^. Formation of the oral microbiome in infancy is influenced by breastfeeding^8,9^, tooth eruption^10^, and introduction of solid foods^11^. Moreover, at the age of 18 months, the oral microbiome is known to become stable and the microbial diversity of tongue is comparable to that of adults^7,12^. Also, vertical transmission of mutans streptococci from parents occurs between the ages of 19 and 31 months^13–15^. This period is called the "window of infection"^16^. Therefore, the transmission of oral bacteria from parents to child is an important factor influencing the formation of oral microbiome in childhood, and it is considered to be one of the factors that changes the oral microbiome into a dysbiotic condition related to early childhood caries onset. However, studies of oral bacteria during this period are often limited to cariogenic bacteria such as *Streptococcus mutans*, and the similarity of the overall microbiome, including commensal bacteria, between parents and child is unknown. Moreover, many reports focus only on mothers and children, and few reports address fathers and children, leaving this latter relationship unclear. A study that involves entire families—mothers, fathers and children—will provide a more complete understanding of how the oral microbiome forms before the “window of infection”.

In this study, we focused on the oral microbiome of 18-month-old infants, at which age deciduous dentition is formed, and the oral microbiome becomes stable, with that of their fathers and mothers. We collected saliva from forty 18-month-old infants and their parents and compared the diversity and composition of the microbiome using next-generation sequencing of 16S rRNA genes.

## Results

### Comparison of oral microbial diversity between infants and adults

**Figure 1:**
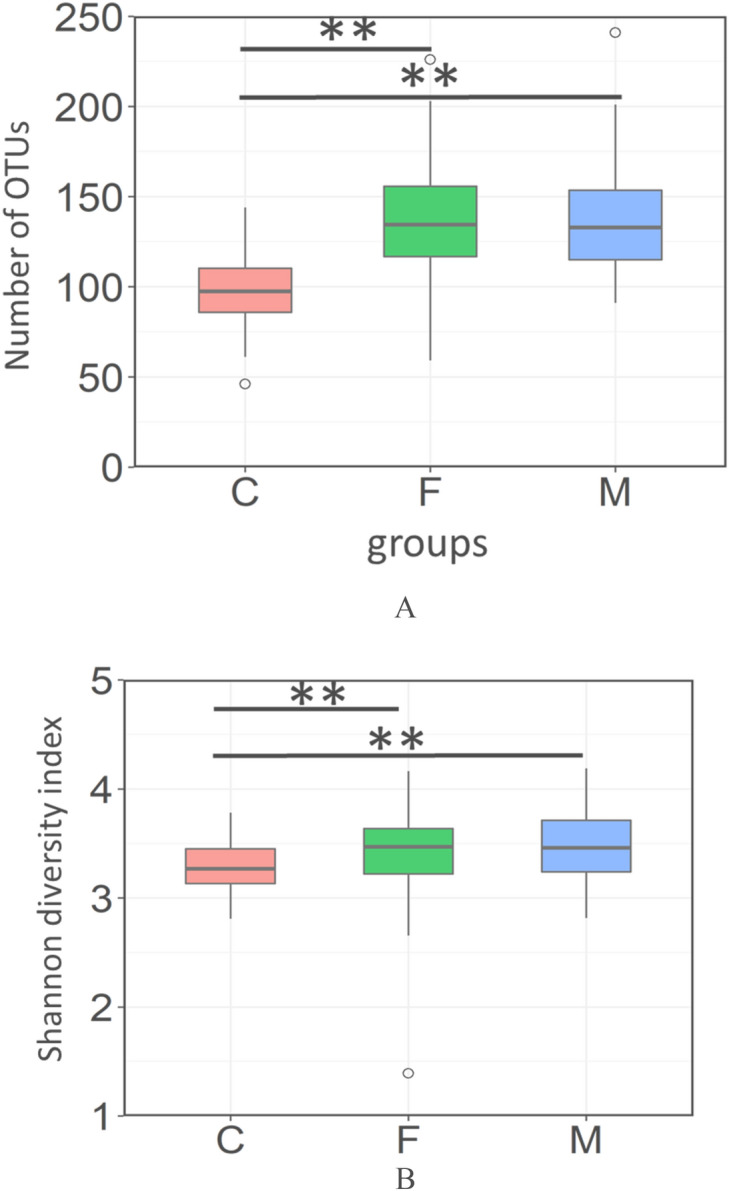
Alpha-diversity of the oral microbiome in each group. Boxplots show the number of observed OTUs (**A**) and the Shannon diversity index (**B**) of each group with the number of sequences rarefied to 3000 reads per sample. *C;* children, *F;* fathers, *M;* mothers. Statistically significant differences are marked with asterisks (Steel–Dwass test, *p < 0.05, **p < 0.01).

We collected saliva from 40 groups of 18-month-old infants and their fathers and mothers. Saliva samples were processed for DNA sequencing and the 16S rRNA region was sequenced using next generation sequencer. First, we compared the diversity of their microbiome. Numbers of detected operational taxonomic units (OTU) and the Shannon diversity index of infant group were significantly lower than those of the parents group (father and mother). No significant differences were observed between the father group and the mother group (Fig. 1, Steel-Dwass test).

### Comparison of oral microbiomes between infants and adults

**Figure 2:**
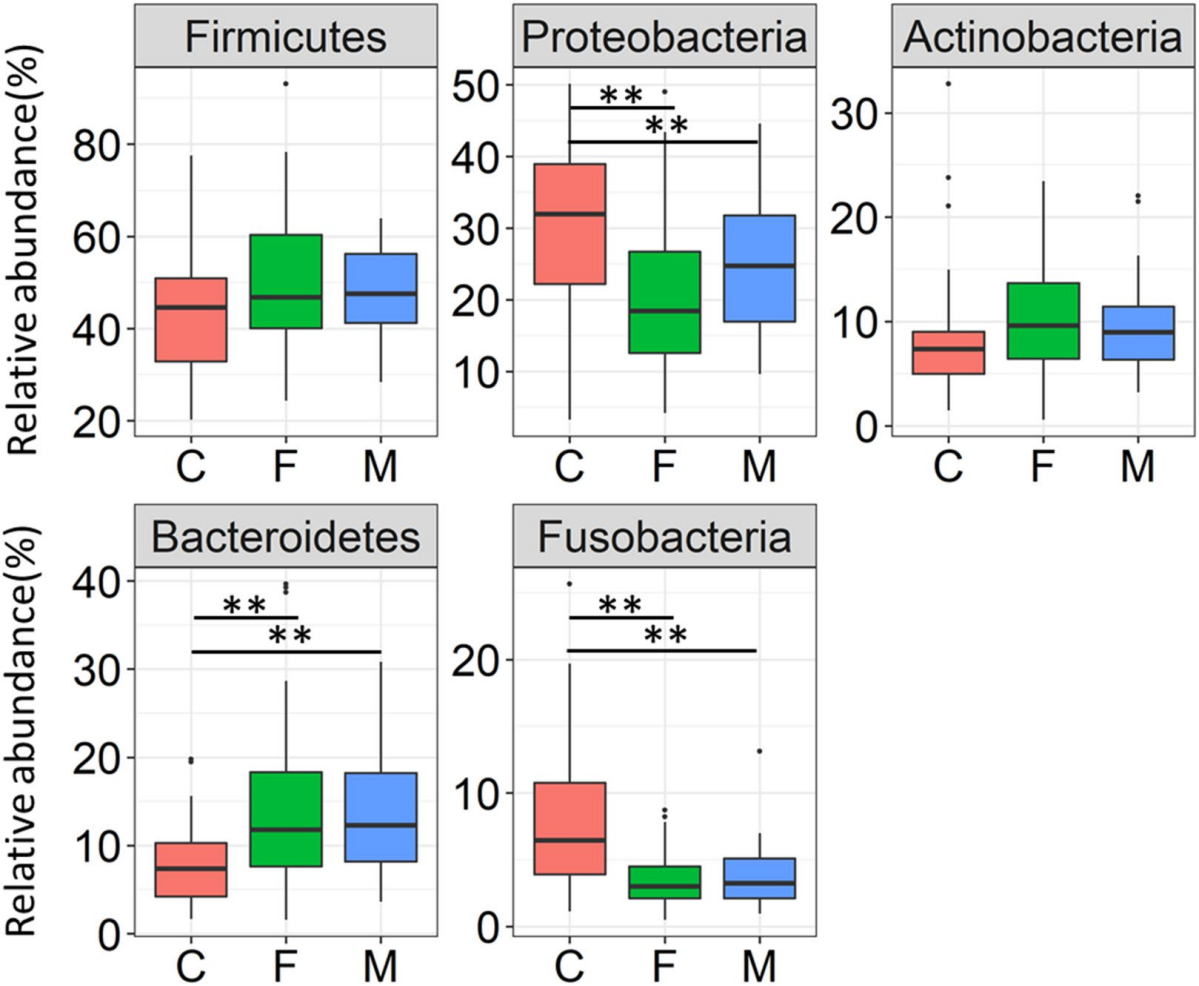
Relative abundance of five predominant phyla in the three groups. The dominant phylum showing more than 1% of the mean relative abundance in the children (C), father (F), and mother (M) groups are shown. Statistically significant differences are marked with asterisks (Steel–Dwass test, *p < 0.05, **p < 0.01).

Subsequently, bacterial composition at the phylum level between 18-month-old infants, father and mother groups were compared. Among the predominant phylum with a > 1% mean relative abundance in each groups, the relative abundance of Proteobacteria and Fusobacteria were significantly higher in infants, whereas the relative abundance of Bacteroidetes was significantly higher in parents (Fig. 2, Steel–Dwass test). As with the microbial diversity, significant differences between father group and mother group were not observed at the phylum level.

### Similarity of microbiomes between infants and adults

**Figure 3:**
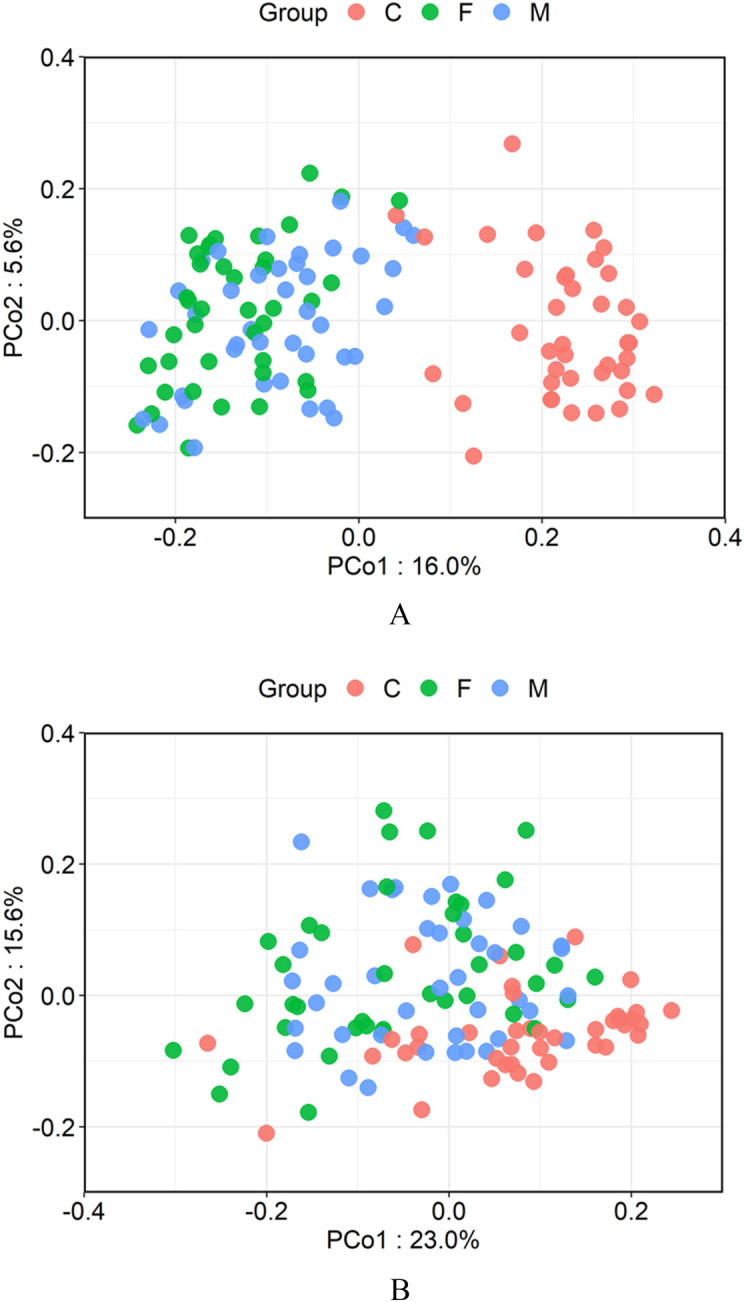
Comparison of the salivary microbiota of the children, fathers, and mothers. (**A**) Unweighted UniFrac-Principal coordinate analysis (PCoA) and (**B**) weighted UniFrac-PCoA of salivary microbiota from the three groups. Samples from children, fathers, and mothers are shown as red, green, and blue, respectively.

The principal coordinate analysis (PCoA) plot based on the UniFrac Distance metric was performed to compare similarities of microbiome between infants and parents (Fig. 3). A permutational multivariate analysis of variance (PERMANOVA) confirmed a significant difference between infants and adults when using either Weighted or Unweighted Distance (*p* < 0.001).

**Figure 4:**
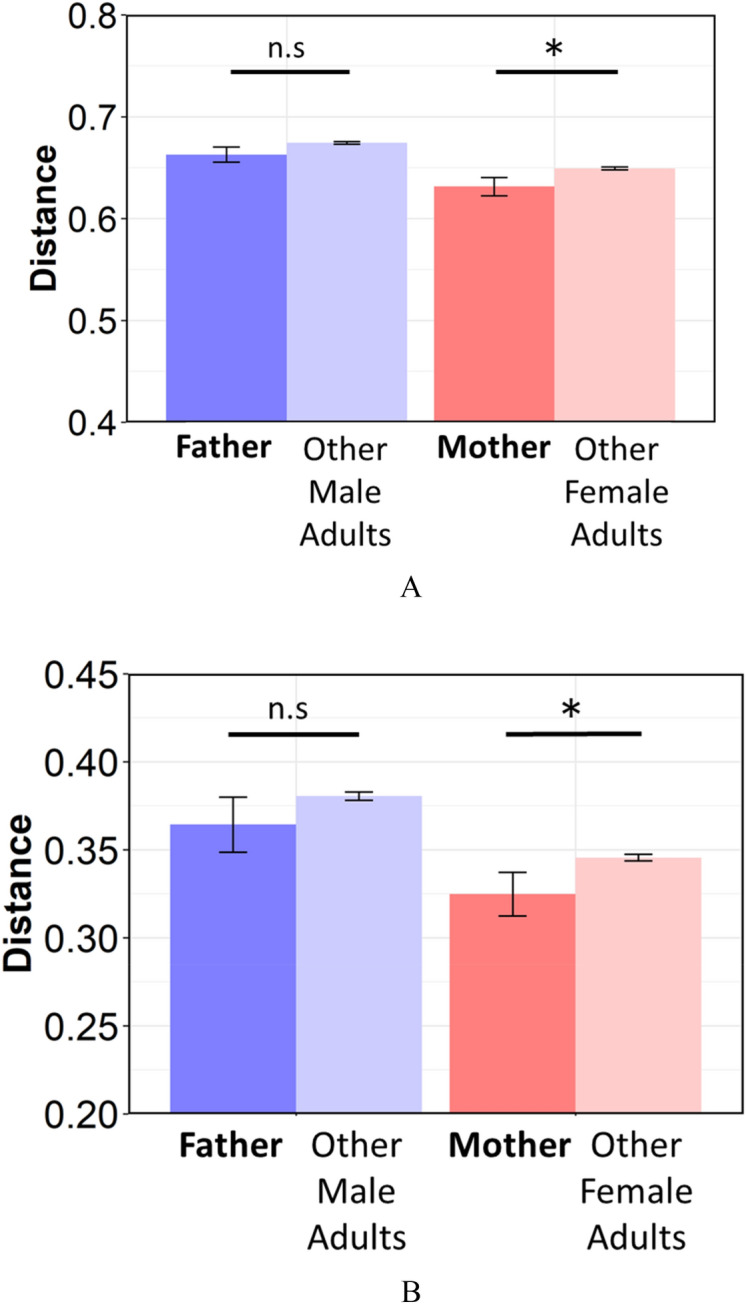
The metrics of (**A**) unweighted, and (**B**) weighted Unifrac Distance between infants and their parents or unrelated adults. *Father;* between infants and their fathers, *other male adults;* between infants and unrelated male adults, *Mother;* between infants and their mothers, *other female adults;* between infants and unrelated female adults. Significant differences are marked with asterisks (*U*-test, *, *p* < 0.05 **, *p* < 0.01) *n.s.* not significant.

Subsequently, to investigate whether an infant's microbiome is more similar to their parents than unrelated adults, comparisons of UniFrac Distance between infants and their parents (father and mother) or the parents of another infant were performed. The similarity between infants and their mothers was significantly higher than the similarity of infants and unrelated female adults in Unweighted and Weighted UniFrac Distance (Fig. 4, *p*-values for both Weighted and Unweighted were 0.03). The similarity between infants and their fathers was also higher than that of infants and unrelated male adults, but differences were not significant (Weighted: *p* = 0.15, Unweighted: *p* = 0.14).

In addition, to examine the relationship of oral microbiome between spouses, we compared the UniFrac Distance between spouses and between unrelated adults. When using either Weighted or Unweighted UniFrac Distance, oral microbiota between spouses showed significantly higher similarity compared with same- or opposite-sex unrelated adults (Supplementaly Fig. S1, *p*-values for both Weighted and Unweighted were less than 0.01). Further, no significant differences in the distance between adults of the same sex or of the opposite sex were found. Thus, no gender differences in adult oral microbiomes were identified.

### OTUs shared by infants and their parents

**Figure 5:**
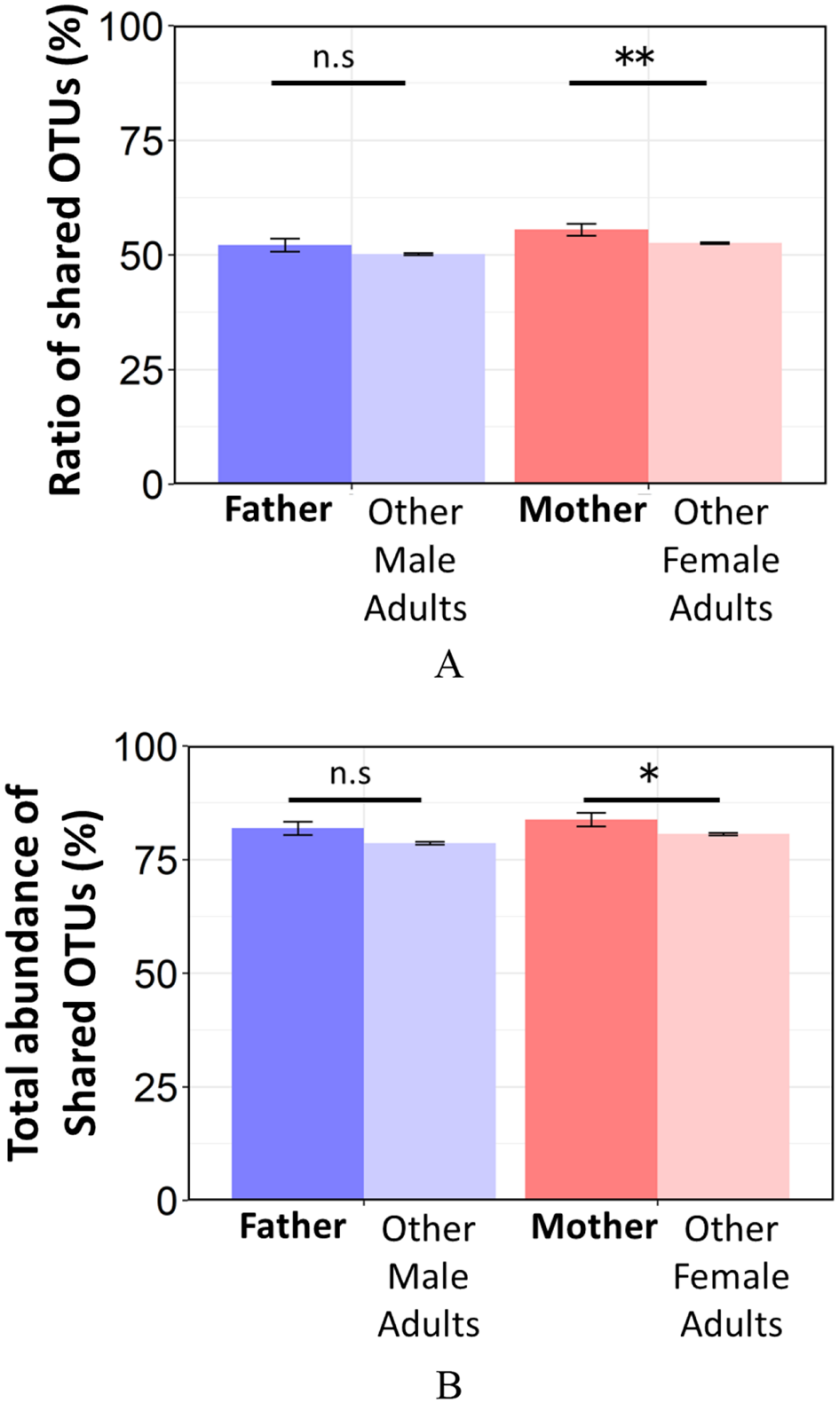
(**A**) Ratio and (**B**) total abundance of OTUs detected in the infant that are shared with their parents or unrelated adults. *Father;* between infants and their fathers, *other male adults;* between infants and unrelated male adults, *mother;* between infants and their mothers, *other male adults;* between infants and unrelated female adults. Significant differences are marked with asterisks (*U*-test, *, *p* < 0.05 **, *p* < 0.01). *n.s.* not significant.

Finally, to determine whether the bacteria detected in the infants were also more frequently present in their parents than in unrelated adults, the ratio and total abundance of OTUs shared by the infants and their parents or unrelated adults were compared (Fig. 5). Similar to the UniFrac distance results, the ratio and total abundance of OTUs shared by infants and their mothers were significantly higher than those shared by infants and unrelated female adults (rate of sharing OTUs: *p* = 0.003, total abundance of sharing OTUs: *p* = 0.04). On the other hand, the ratio and total abundance of OTUs shared by infants and their fathers were not significantly higher than those shared by infants and unrelated male adults (rate of sharing OTUs: *p* = 0.16, total abundance of sharing OTUs: *p* = 0.11). OTUs highly shared between infant and their parents, were assigned to genus such as *Granulicatella, Streptococcus, Veillonella*, *Neisseria*, *Haemophilus*, *Rothia*, and *Fusobacterium*. Table 1 shows a list of OTUs that are highly (> 80%) shared between infants and their parents. Furthermore, OTUs assigned to typical cariogenic and periodontal pathogens (*S. mutans* and Red Complex (*Porphyromonas gingivalis*, *Tannerella forsythia*, and *Treponema denticola*)) were not detected in infants, with the exception of *S. mutans* which was detected in only one infant (Supplementary Table S1).

**Table 1:** Highly (> 80%) shared OTUs between infant and their parents.

	Corresponded Species	Identity (%)	Detection rate (%)			Sharing rate (%)	
			Father	Mother	Infant	Father–Infant	Mother–Infant
OTU0030	*Granulicatella para-adiacens*	100	100	100	100	**100**	**100**
OTU0038	*Rothia mucilaginosa*	100	100	100	100	**100**	**100**
OTU0209	*Haemophilus parainfluenzae*	98.71	100	100	100	**100**	**100**
OTU0348	*Streptococcus sp. M334*	98.09	100	100	100	**100**	**100**
OTU0446	*Veillonella parvula*	98.78	100	100	100	**100**	**100**
OTU0826	*Streptococcus genomosp. C5*	99.04	95	100	100	*95*	**100**
OTU0041	*Neisseria mucosa*	100	100	97.5	100	**100**	*97.5*
OTU0005	*Veillonella dispar*	99.39	100	100	97.5	*97.5*	*97.5*
OTU0188	*Fusobacterium nucleatum*	98.98	100	97.5	97.5	*97.5*	*97.5*
OTU0207	*Streptococcus mitis*	98.09	100	100	97.5	*97.5*	*97.5*
OTU0108	*Rothia aeria*	100	97.5	100	97.5	*95*	*97.5*
OTU0082	*Lautropia mirabilis*	100	95	100	97.5	*92.5*	*97.5*
OTU0113	*Porphyromonas CW034*	99.37	92.5	100	97.5	*92.5*	*97.5*
OTU0512	*Streptococcus oralis*	97.45	97.5	97.5	97.5	*95*	*95*
OTU0004	*Streptococcus sanguinis*	100	97.5	100	95	*92.5*	*95*
OTU0276	*Fusobacterium nucleatum*	99.68	90	97.5	97.5	*90*	*95*
OTU0488	*Gemella haemolysans*	98.43	82.5	97.5	97.5	*80*	*95*
OTU0043	*Gemella sanguinis*	100	100	97.5	95	*95*	*92.5*
OTU0264	*Granulicatella para-adiacens*	97.48	97.5	95	97.5	*95*	*92.5*
OTU0003	*Streptococcus salivarius*	99.68	100	100	92.5	*92.5*	*92.5*
OTU0079	*Neisseria sicca*	100	95	95	97.5	*92.5*	*92.5*
OTU0021	*Actinomyces odontolyticus*	99.35	97.5	100	92.5	*90*	*92.5*
OTU0430	*Gemella haemolysans*	97.18	90	90	100	*90*	*90*
OTU1011	*Gemella haemolysans*	98.75	90	92.5	97.5	*87.5*	*90*
OTU0009	*Streptococcus oralis*	99.36	92.5	97.5	92.5	*85*	*90*
OTU1189	*Streptococcus mitis*	97.47	90	95	95	*85*	*90*
OTU1019	*Haemophilus parainfluenzae*	97.1	97.5	95	92.5	*92.5*	*87.5*
OTU0592	*Streptococcus infantis*	98.1	97.5	97.5	87.5	*85*	*87.5*
OTU0882	*Streptococcus mitis*	98.73	95	95	90	*85*	*87.5*
OTU0040	*Gemella haemolysans*	98.12	90	97.5	90	*82.5*	*87.5*
OTU0073	*Streptococcus sp. oral taxon 058*	99.36	90	92.5	90	*82.5*	*85*
OTU0190	*Streptococcus infantis*	98.73	97.5	100	85	*82.5*	*85*
OTU0008	*Haemophilus sp. CCUG 17210*	99.35	85	92.5	90	77.5	*85*
OTU0141	*Bergeyella 602D02*	99.68	77.5	90	92.5	70	*82.5*
OTU1362	*Streptococcus mitis*	98.09	95	90	90	*85*	*80*
OTU0051	*Streptococcus oralis*	98.73	95	92.5	87.5	*82.5*	*80*
OTU0007	*Haemophilus parainfluenzae*	99.68	95	90	85	*80*	77.5

Subsequently, the relationship between indicators known to be related to oral microbiome formation, such as the introduction of baby food, the induction of deciduous tooth eruption, and the mode of feeding (breast milk/artificial milk), and the similarity of the oral microbiome between the infants and parents were analyzed, and none of the indexes exhibited a clear relationship (Supplementary Table S2). Moreover, whether or not the infant was weaning did not affect the similarity between the infant and their parents (Supplementary Table S3). Circadian rhythm has also been described to contribute to the salivary microbiome^17^; however, we did not see significant relationship between the sampling time and similarity of the microbiome between the infants and their parents (Supplementary Table S2).

## Discussion

In this study, we attempted to clarify the relationships of the oral microbiome in 18-month-old infants and their parents. We collected saliva from forty 18-month-old infants and their parents and compared diversity and composition of their microbiome using next-generation sequencing of 16S rRNA genes. We showed that diversity and composition of oral microbiome in 18-month-old infants differs from their parents. Meanwhile, the oral microbiome of the infants was more similar to their mothers than that of unrelated female adults. The bacteria highly shared between infants and parents included not only commensal bacteria but also disease related bacteria. These results suggested that the oral microbiome of the mother influences the oral microbiome of their children by 18 months.

In this study, the oral microbiota of 18-month-old infants were less diverse than that of adults (Fig. 1). This result was consistent with studies comparing the salivary microbiome in infants and adults, from the infants’ birth to 5 years of age^18,19^. These results suggested that the oral microbiome of the infants was still immature at 18 months of age. Moreover, another study reported that the microbial diversity of the tongue in 18-month-old infants is comparable to that of adults^7^. These results suggest that the maturity of the oral microbiome varies depending on the location in the oral cavity.

Significant similarity in microbiomes was found in infants and their mothers compared with unrelated adults (Fig. 4). The ratio and total abundance of OTUs shared by infants and their mothers were significantly higher than those shared by infants and unrelated female adults (Fig. 5). Considering these results, the oral microbiome of parents influences the oral microbiome of infants and both commensal and pathogenic oral bacteria may be transmitted from parents to their infants before the “window of infection”.

It has been reported that oral cavity microbiota differs between caries active and healthy children, and the shift from symbiotic microbiome to dysbiotic microbiome is linked to early childhood caries onset^18,20,21^. Regarding the factors causing the shift to dysbiotic microbiome, an increased frequency of sugar intake is mainly focused on. However, it was also reported that feeding habits are not significantly different between children with or without severe early childhood caries^21^, and the transmission of oral bacteria from parents is also considered to be one of the important factors for oral microbiome shift^22,23^. In this study, most of the bacteria highly shared by infants and their parents were oral commensal bacteria such as *Streptococcus*, *Veillonella*, *Neisseria*, *Haemophilus*, and *Rothia*. However, OTUs assigned to *Fusobacterium nucleatum*, which is involved in periodontal disease^24,25^, were also shared at a high rate. (Table 1). *F. nucleatum* has been suggested to play an important role in multispecies dental biofilm formation due to its ability to adhere to a very large variety of different microorganisms^26^. Consequently, *F. nucleatum* is considered to be associated with the transition from a commensal community to a pathogenic community^27,28^. Thus, the oral microbiome of parents may influence the shift of oral microbiome of their children from symbiotic to dysbiotic microbiome.

In contrast, no significant similarity was detected between infants and their fathers compared with the infant and unrelated adults, suggesting that mothers' oral microbiota have a greater effect on the formation of their children's oral microbiome than fathers'. This finding may be due to more intimate contact with the mother's microbiome, such as during breastfeeding.

In addition, oral microbiome between spouses was significantly more similar than microbiomes between unrelated adults (Supplementaly Fig. S1). This result suggests that even between individuals that are genetically different, similar oral microbiomes are formed likely by activities such as kissing and contact with same microbial sources or diets. Kissing causes a temporary exchange of oral bacteria^29^ and *Porphyromonas gingivalis,* a typical periodontal pathogen, is known to be transmitted between spouses^30–32^. Although we did not obtain lifestyle information such as kissing from the subjects in this study, it is simply assumed that spouses' physical contact is one explanation for similarity of oral microbiome between spouses.

The limitation of this study is that analysis using 16S rRNA is not suitable for detailed bacterial type identification. More detailed analysis, such as shotgun metagenomics, must be used to determine if transmission of oral bacteria occurs among family members. In this study, we could not obtain information on the history of caries, periodontal disease, smoking, or systemic diseases (such as inflammatory bowel disease) that are known to affect the oral microbiome^33,34^. Therefore, further studies are warranted to understand the effects of these factors on the similarity of the oral microbiome between infants and their parents.

In conclusion, a comparison of oral microbiomes of 18-month-old infants and their parents revealed that oral microbiomes were significantly similar between infants and their mothers compared with unrelated adults. The bacteria highly shared between infants and parents included not only commensal bacteria but also disease related bacteria. These results suggested that the oral microbiome of the parents influences that of their children before the window of infection. Hence, it is important for parents to control their oral microbiome by continuous professional treatment and self-care, and if they are suffering from oral diseases, should be careful of infections in their children.

## Methods

### Study subjects

We recruited forty 18-month-old infants (19 boys and 21 girls) and their biological fathers [aged 26–48 years (mean ± s.d., 34.8 ± 4.9 years)] and mothers [aged 27–42 years (mean ± s.d., 33.2 ± 3.7 years)] who were living together. The fathers and/or mothers were employees or employee’s family of a manufacturer located in Tokyo, Japan. Children had not received antibiotics in the six months before the collection of samples. We examined the age of introduction of baby food, induction of deciduous tooth eruption, weaning, and ratio of breast milk/artificial milk. These data are listed in Supplementary Table S4. All adult participants understood the purpose of the study and provided informed consent. Because our affiliated institutions did not have any ethics committee, this study was given ethical approval by ethics committee of the academic society (Ethics committee of the Japanese Society for Oral Health, Tokyo, Japan, Issuing number: No. 26-5). Although authors are members of this academic society, none of us are affiliated to its ethics committee. All experiments were performed in accordance with approved guidelines.

### Sample collection

Before sample collection, participants were instructed not to brush their teeth from the last meal to the time of sampling and were prohibited from eating or drinking for at least 1 h before sampling. Infant saliva was collected using SalivaBio Infant's Swabs (Salimetrics, US, CA)^35^. Saliva of infants were collected by sucking saliva accumulated in the oral cavity using a swab stick. Parent’s saliva samples were collected as mouth-rinsed water^36^. Briefly, participants rinsed their mouth vigorously with 3 mL sterilised water for 10 s, and then spat into a sterilised specimen tube. All samples were stored at refrigerated condition and centrifuged at 16,400 × *g* for 5 min within 30 h after collection. Resulting pellets were stored at − 80 °C until DNA extraction.

### DNA extraction and sequencing of 16S rRNA gene amplicons

Genomic DNA was isolated from the collected samples using a Nexttec 1-Step DNA Isolation Kit (nexttec Biotechnologie GmbH, Leverkusen, Germany). PCR used universal primers (27Fmod and 338R) for 16S rRNA gene sequencing, as previously described^36,37^. PCR used Ex Taq polymerase (Takara Bio, Shiga, Japan) and approximately 20 ng of template DNA.

Thermal cycling was performed in a Veriti Thermal Cycler (Life Technologies Japan, Tokyo, Japan). Cycling conditions were: initial denaturation at 96 °C for 2 min, followed by 25 cycles of denaturation at 96 °C for 30 s, annealing at 55 °C for 45 s, extension at 72 °C for 1 min, and final extension at 72 °C. PCR amplicons were purified using AMPure XP magnetic purification beads (Beckman Coulter, CA, USA) and quantified using a Quant-iT PicoGreen dsDNA Assay Kit (Life Technologies Japan). After quantification, mixed samples were prepared by pooling approximately equal amounts of each amplified DNA. Samples were sequenced using a MiSeq Reagent Kit V3 (300 × 2 cycles) and a MiSeq sequencer (Illumina, CA, USA), following the manufacturer's instructions.

### Data processing

We used an analysis pipeline for processing the 16S rRNA gene V1–V2 region, as previously reported^34,38^. Briefly, after multiplexed sequencing of the 16S amplicons, sequences were assigned to samples based on their barcode sequences. Reads with an average quality value < 25, inexact matches to both universal primers, and possible chimeric reads were eliminated. Among high-quality reads, 3000 reads per sample were randomly chosen and used for the comparative microbiome analysis. We sorted selected reads with the average quality value and grouped them into OTUs using the UCLUST (v.5.2.32) algorithm with a 97% identity threshold^39^. Taxonomic assignments for each OTU were made by similarity searching against publicly available 16S database using the GLSEARCH program (v.36.3.8 g). The 16S database was constructed from three publically available databases, as previously described^19^: Ribosomal Database Project (RDP) v.10.31, CORE (http://microbiome.osu.edu/ (31 January 2017, date last accessed)), and the reference genome sequence database obtained from the NCBI FTP site (ftp://ftp.ncbi.nih.gov/genbank/ (December 2011, date last accessed)). For assignment at the phylum levels, sequence similarity thresholds of 70% were applied, respectively^39^. All high-quality 16S V1–V2 sequences were submitted to the DDBJ/GenBank/EMBL database (Accession number DRA010385).

### Data analysis

We used Mann-Whitney *U*-test and Steel-Dwass test where appropriate for comparisons of categorical variables. We also used UniFrac distance^40^ for dissimilarity (distance) assessment between pairs of samples. PCoA was used to visualise similarities/dissimilarities in microbiome structures from the UniFrac Distance. We conducted a PERMANOVA to compare overall microbiome structures. Differences at *p* < 0.05 were considered statistically significant. All analyses were performed using R software program (v3.4.3).

## Supplementary information

Supplementary Information.
