# Supplementary material for: Comparison of oral microbiome profiles in 18-month-old infants and their parents
Source: Sci Rep. 2021 Jan 13;11:861. doi: 10.1038/s41598-020-78295-1 (PMC7806650; doi:10.1038/s41598-020-78295-1)
Supplement: Supplementary file 1 — Supplementary Information. [file 41598_2020_78295_MOESM1_ESM.pdf]

Comparison of oral microbiome profiles in 18-month-old infants and their parents

Ryutaro Jo[1,*], Kazuma Yama[1], Yuto Aita[1], Kota Tsutsumi[1], Chikako Ishihara[1], Masato Maruyama[1], Kaori Takeda[2], Eiji Nishinaga[1], Ken-ichiro Shibasaki[1], Seiji Morishima[2]

1. Research and Development Headquarters, Lion Corporation. 7-2-1 Hirai, Edogawa-ku, Tokyo, 132-0035, Japan.
2. The Lion Foundation for Dental Health. 1-3-7, Honjo, Sumida-ku, Tokyo, 130-8644, Japan.

*Correspondence:

Ryutaro Jo

Research and Development Headquarters, Lion Corporation. 7-2-1 Hirai, Edogawa-ku, Tokyo, 132-0035, Japan.

E-mail: r-jo@lion.co.jp

TEL: (+81) 3-3621-4518

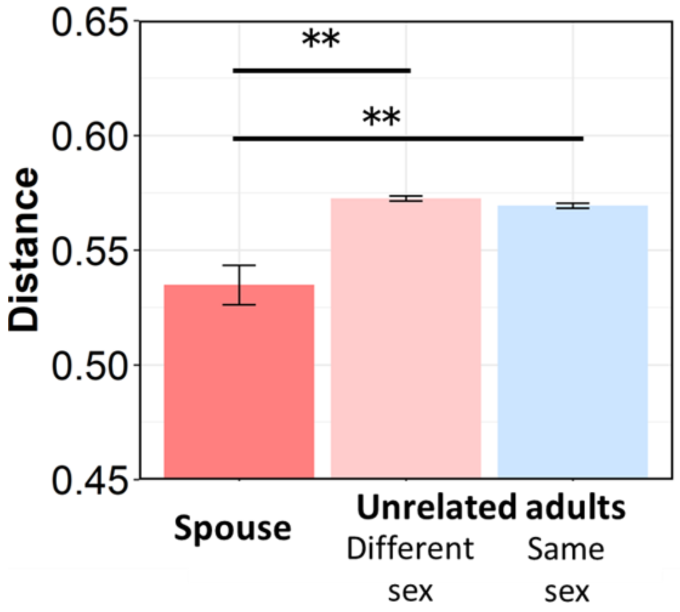

Supplementary Figure S1A

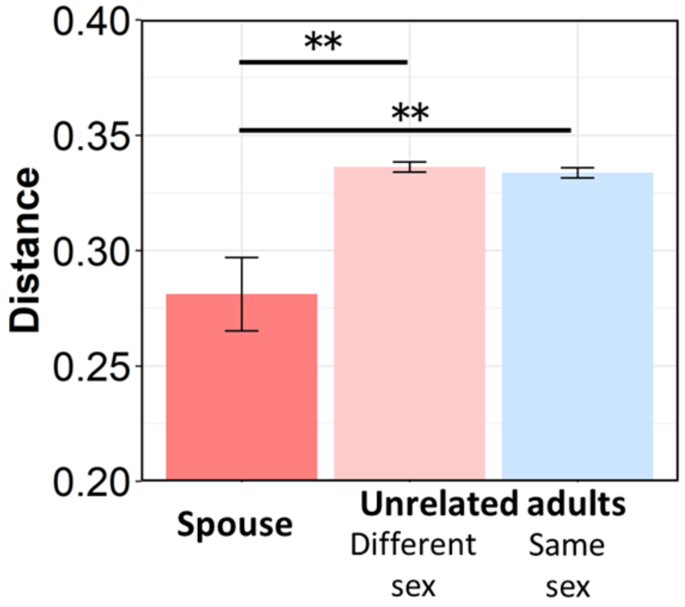

Supplementary Figure S1B

Supplementary Figure. S1. The metrics of (A) unweighted, and (B) weighted Unifrac Distance between spouses or unrelated adults. Spouses; between spouses. Unrelated adults Different sex; between unrelated (not spouses) different sex adults, Unrelated adults Same sex; between unrelated (not spouses) same sex adults. Significant differences are marked with asterisks (Steel‐Dwass test, *, $p < 0.05$ **, $p < 0.01$)

Supplementary Table S1. Detection and sharing rate of OTUs that identified as pathogens between infant and their parents. OTUs that are >97% homologous to the pathogen were listed.

	Corresponded Species	Identity (%)	Detection rate (%)			Sharing rate (%)	
			Father	Mother	Infant	Father-Infant	Mother-Infant
OTU00118	*Streptococcus mutans*	99.69	30	17.5	2.5	2.5	0
OTU00435	*Porphyromonas gingivalis*	99.68	22.5	7.5	0	0	0
OTU00388	*Tannerella forsythia*	99.68	47.5	27.5	0	0	0
OTU00278	*Treponema denticola*	99.68	30	20	0	0	0

Supplementary Table S2. Spearman correlation coefficient between the indicators known to be related to the oral microbiome and similarity of oral microbiome between infants and their parents. Weighted F&C: weighted unifrac distance between fathers and their children, Weighted M&C: weighted unifrac distance between mothers and their children, unweighted F&C: unweighted unifrac distance between fathers and their children, Unweighted M&C: unweighted unifrac distance between mothers and their children, shared OTUs ratio F&C: ratio of OTUs detected in the infants that are shared with their fathers, shared OTUs ratio M&C: ratio of OTUs detected in the infants that are shared with their mothers, shared OTUs abundance F&C: total relative abundance of OTUs detected in the infants that are shared with their fathers, shared OTUs abundance F&C: total relative abundance of OTUs detected in the infants that are shared with their mothers.

	Introduction of baby food (Months)	Induction of deciduous tooth eruption (Months)	Ratio of Breast milk/ Artificial milk from birth to 12 months	Sampling Time (Hour)
Weighted F&C	−0.230	−0.242	0.132	0.008
Weighted M&C	0.017	−0.168	0.023	−0.168
Unweighted F&C	−0.208	−0.045	−0.184	−0.016
Unweighted M&C	0.084	0.047	−0.135	−0.057
Ratio of shared OTUs F&C (%)	0.253	0.147	0.136	0.082
Ratio of shared OTUs M&C (%)	0.088	0.269	0.115	0.203
Total abundance of shared OTUs F&C (%)	0.148	0.011	0.038	−0.074
Total abundance of shared OTUs M&C (%)	0.144	0.161	0.091	−0.220

Supplementary Table S3. Comparison of microbial diversity and similarity of oral microbiome between infants and their parents with or without weaning. Weighted F&C: weighted unifrac distance between fathers and their children, weighted M&C: weighted unifrac distance between mothers and their children, unweighted F&C: unweighted unifrac distance between fathers and their children, unweighted M&C: unweighted unifrac distance between mothers and their children, shared OTUs ratio F&C: ratio of OTUs detected in the infants that are shared with their fathers, shared OTUs ratio M&C: ratio of OTUs detected in the infants that are shared with their mothers, shared OTUs abundance F&C: total relative abundance of OTUs detected in the infants that are shared with their fathers, shared OTUs abundance F&C: total relative abundance of OTUs detected in the infants that are shared with their mothers. Each value is indicated as mean ± standard deviation.

	Weaning (n = 29)	Not weaning (n = 11)	*p*-value (*U*-test)
Weighted F&C	0.36±0.11	0.37±0.08	0.835
Weighted M&C	0.32±0.07	0.32±0.09	0.473
Unweighted F&C	0.66±0.04	0.67±0.05	0.419
Unweighted M&C	0.62±0.05	0.65±0.06	0.511
Ratio of shared OTUs F&C (%)	50.8±9.6	54.3±6.6	0.739
Ratio of shared OTUs M&C (%)	55.2±7.6	56.0±8.7	0.952
Total abundance of shared OTUs F&C (%)	80.9±10.1	83.6±6.5	0.988
Total abundance of shared OTUs M&C (%)	84.9±7.5	81.9±11.6	0.188

Supplementary Table S4. Summaries of information on infants

Gender (Boys/Girls)	19 / 21
Weaning (Yes / No)	29 / 11
	Median (Min-Max)
Ratio of Breast milk/ Artificial milk (%)	75 (0 – 100)
Introduction of baby food (Months)	5.5 (5.0 – 6.7)
Induction of deciduous tooth eruption (Months)	7.0 (4 – 12)
